# A Rare Case of Non-Obstructive Membrane of the Body of Left Atrial Appendage Incidentally Found in Asymptomatic Adult Woman

**DOI:** 10.4021/cr148w

**Published:** 2012-01-20

**Authors:** Bong Gun Song, Gu Hyun Kang, Yong Hwan Park, Woo Jung Chun, Ju Hyeon Oh

**Affiliations:** a Division of Cardiology, Cardiac and Vascular Center, Department of Medicine, Samsung Changwon Hospital, Sungkyunkwan University School of Medicine, Changwon, Korea

**Keywords:** Membranes of left atrial appendage, Echocardiography

## Abstract

The membranes of the left atrial appendage cavity are very rare entity and their clinical significance is not clear. We reported a rare case of a non-obstructive membrane traversing the body of the left atrial appendage incidentally found in asymptomatic adult woman.

## Introduction

The membranes of the LAA cavity are very rare entity [1]. The origin of these membranes involving the LAA and their clinical significance is not clear [1]. Here, we described a rare case of non-obstructive membrane traversing the body of the left atrial appendage incidentally found in asymptomatic adult woman.

## Case Report

**Figure 1:**
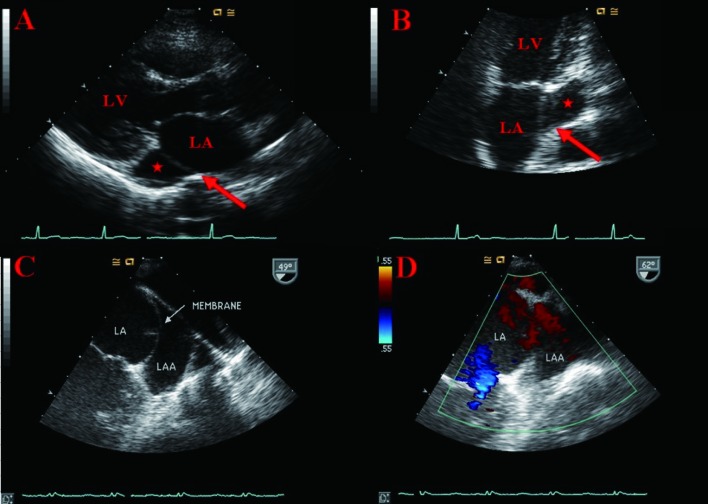
TTE showed left atrium with a linear, mobile, membrane-like structure (arrow) across left atrial appendage (asterisk) (A, B). TEE demonstrated a linear, mobile, membrane-like structure traversing the body of left atrial appendage without a turbulent Doppler color flow across the membrane with Color Doppler (C, D). (LA, left atrium; LV, left ventricle; LAA, left atrial appendage).

A 50-year-old woman was referred to our cardiology outpatient department for further evaluation of abnormal trans-thoracic echocardiogram (TTE) findings found during a routine health examination program. The patient denied having any diseases in the past. She did not have any symptoms and her physical examination was normal. Upon her visit to the hospital, the patient’s blood pressure was 120/80 mmHg, with a regular pulse of 82 beats/min. A 12-lead electrocardiogram showed normal sinus rhythm. Laboratory tests revealed no significant abnormalities. TTE showed the normal left ventricular dimension and function. TTE on the parasternal long axis and apical 2 chamber views showed left atrium (LA) with a linear, mobile, membrane-like structure across the left atrial appendage (LAA) (Fig. 1A, B). Trans-esophageal echocardiogram (TEE) demonstrated a linear, mobile, membrane-like structure traversing the body of the LAA (Fig. 1C). A turbulent Doppler color flow jet with a mosaic pattern was not seen through a linear, mobile, membrane-like structure (Fig. 1D) and velocity step-up across the membrane was not observed in Pulsed-wave Doppler. No thrombus or spontaneous echo contrast was found in the LA and LAA. Since the patient was asymptomatic, the initial decision of the patient care was to follow-up the patient clinically.

## Discussion

The membranes of the LAA cavity are very rare entity [1]. The origin of these membranes involving the LAA is not clear. The most likely explanation of the origin of these membranes would appear to be a congenital anatomic variation [1-3]. In five reports [1, 2], non-obstructive membranes located in the body of the LAA have been described and in two cases [4, 5] obstructive membranes at the opening of the LAA, causing functional stenosis have been reported. Similar to previous reports [1, 2], we described a case that has a thin mobile membrane-like structure at the body the LAA. It does not cause an obstruction at the LAA opening as demonstrated by absence of velocity step-up on pulse-wave Doppler of the LAA and by a lack of turbulence with color flow Doppler. The different diagnosis of linear structures within LAA cavity may include prominent pectinate muscles, side lobe artifacts, partial resorption of prior LAA thrombi and localized pericardial effusion [1, 2]. The clinical significance of this membrane has not been known [2]. Previous reports on incomplete surgical ligation or recanalization of the LAA have emphasized the potential for stagnant flow within the LAA and possible thrombus formation with systemic embolization [1, 2]. Though speculative, this scenario is unlikely with non-obstructive membranes because stagnant flow localized distal to membrane was not demonstrated.

## References

[1] Bakris N, Tighe DA, Rousou JA, Hiser WL, Flack JE, Engelman RM (2002). Nonobstructive membranes of the left atrial appendage cavity: report of three cases. J Am Soc Echocardiogr.

[2] Correale M, Ieva R, Deluca G, Di Biase M (2008). Membranes of left atrial appendage: real appearance or "pitfall". Echocardiography.

[3] Ernst G, Stollberger C, Abzieher F, Veit-Dirscherl W, Bonner E, Bibus B, Schneider B (1995). Morphology of the left atrial appendage. Anat Rec.

[4] Coughlan B, Lang RM, Spencer KT (1999). Left atrial appendage stenosis. J Am Soc Echocardiogr.

[5] Ha JW, Chung N, Hong YS, Cho BK (2001). Left atrial appendage stenosis. Echocardiography.

